# Inequality in dental care expenditure in Iranian households: analysis of income quintiles and educational levels

**DOI:** 10.1186/s12903-021-01912-6

**Published:** 2021-10-26

**Authors:** Elahe Najafi, Mostafa Amini-Rarani, Maryam Moeeni

**Affiliations:** 1 School of Management and Medical Information, Isfahan University of Medical Sciences, Isfahan, Iran; 2 Social Determinants of Health Research Center, Isfahan University of Medical Sciences, Isfahan, Iran; 3 Health Management and Economics Research Center, Isfahan University of Medical Sciences, Isfahan, Iran

**Keywords:** Dental care, Health expenditure, Health care inequalities, Gini coefficient, Concentration index

## Abstract

**Background:**

Oral health is a major public health issue which affects the human life. Access to dental care is one of the important factors in maintaining oral health. This study was aimed to investigate inequality in dental care expenditure in Iranian households.

**Methods:**

The present study is a secondary analysis of a national cross-sectional survey. The data collected from the Households Income and Expenditure Survey in 2016 and 2017. The final sample consisted of 54,354 households living in rural and urban regions of all the provinces. Inequalities in household’s dental care expenditure per capita in respect to income quintiles and educational level were measured based on the Gini coefficient and concentration index.

**Results:**

The results showed that about 8% of households had paid for dental care during the month before sampling. The Gini coefficient value was estimated to be 0.97 and 0.96 for dental care expenditure per capita respectively in absolute and relative measure. It indicated a significant inequality in the dental expenditure among the sample households. The values of concentration index were positive and significant for all dental care subcategories in respect to the provincial and national income quintiles as well as the educational level of the head of the household.

**Conclusions:**

Income and educational inequality in the both absolute and relative dental services expenditure of the Iranian households were in favor of higher income groups as well as higher educational level of household heads. Income inequality was higher in total dental care expenditure per capita and all its subcategories than the educational inequalities of dental expenditure. In order to reduce these inequalities, the policymakers need to pay special attention to low-income households, particularly those with low-educated heads.

## Background

Oral health is a major public health issue which affects the human life [1]. According to World Health Organization, oral health can be defined as lack of pain and sores, mouth and throat cancer, mouth infections and ulcers, periodontal disease, tooth decay, tooth loss, and other diseases and disorders that limit the ability of the patient to bite, chew, smile, and talk [2]. Disorders such as deciduous and permanent tooth decay, periodontal diseases, and loss of decayed teeth affect the well-being and health of a large number of people worldwide. In recent decades, oral health has been promoted in many communities [3]. Over the past 50 years, significant progress has been made in oral health in high-income and most of middle-income countries, but it is not the case in low-income countries [4].

Access to dental care is one of the important factors in maintaining oral health. Still, some people do not access to needed dental cares. As an example, oral and dental health status of children and elderly, as the two high-risk groups, is continuously studied in most countries [5–9]. Overall, 60–90% of students and 100% of adults in the world have dental caries. Additionally, to WHO’s report, the ratio of adults aged 65–74 years old with dental and oral problems was 40% in low-income countries and 30% in high-income countries. But, the ratio of people who received dental health services was 30% and 75% in low- and high-income countries, respectively [10].

In recent years, some studies on socio-economic inequality in oral health has been conducted. In those studies, inequalities in oral health have been recognized based on socio-economic status (SES), such as income level, education, occupational status, and place of residence in many countries [11–14]. In these studies one of the main reasons for inequality in oral health is the low socio-economic status. For example, a study conducted in South Korea showed inequalities in oral health in respect to income status, educational level, and occupation [11, 15]. According to the study of Peltzer et al. (2014), poor oral health is worse in low-income and middle income countries than in high-income countries. And, Shekar et al. (2011) reported a higher average of untreated tooth decay and tooth loss among poor [16]. Additionally, Di Bella (2017) showed that various economic and social factors lead children, elderly, rural residents, homeless people, and low-income people to be severely affected by dental diseases, such as dental cavities and periodontitis [17]. Results of the study by Ravaqi et al. (2013) demonstrated that economic inequality significantly affected oral health. In the same vein, inequality to dental care access in respect to socio-economic status has been evident [5]. A number of studies in this scope are based on measure of statistical dispersion. Some related Studies have been conducted by Cornejo-Ovalle et al. (2015) in Chilean adults [6], Palència et al. (2014), and Listl (2011) in European people aged ≥ 50 years [7, 8], Bhandari et al. (2015) in a sample of individuals in OECD countries, and Homaie Rad et al. (2016) in a sample of people in Shiraz, Iran [9, 18].

To the best of our knowledge, no study has been conducted in Iran to determine the inequality in dental care expenditure in Iranian households based on national surveys including big data. Thus, this study is aimed to investigate inequality in dental care expenditure in Iranian households based on the data of Households Income and Expenditure Survey (HIES). Considering high generalizability of this survey which includes national big data, results of the study could provide health policymakers in Iran with a better image of the patterns of dental care expenditure.

## Methods

The present study is a secondary analysis of the cross-sectional data collected from the HIES in 2016 and 2017. The HIES’s data which had being gathered from all the provinces formed the primary sample. The number of urban households was 18,809 and 18,701 in 2016 and 2017, respectively. These figures for rural households were 19,337 and 19,261 in 2016 and 2017, respectively. The only criterion for including households into the study sample was providing complete responses to the relevant questions. In total, a number of 21,754 households were excluded due to their incomplete relevant information. Therefore, the final sample consisted of 54,354 households.

In the first stage, the dataset in the Access files were processed and converted to a final data file. These files included demographic information of households, non-health care expenditure, health care expenditure, and all kinds of household income.

In the second stage, the variables of dental care expenditure, income quintiles, and educational level, were extracted from available data. In the HIES questionnaire, the households had been asked about their dental care expenditure during the month before the interview. In the questionnaire, several codes are defined for dental care, which include the number of visits, extraction, scaling, dental surgery, root canal treatment, periodontal surgery, dental implants and prostheses, and orthodontic services. Therefore, the data related to households’ dental care expenditure were extracted by the mentioned codes. We calculated household’s dental care expenditure per capita in absolute and relative measures. The absolute measure of household’s dental care expenditure per capita was calculated from the ratio of total household expenditure to household size. The relative measure was absolute measure of household’s dental care expenditure proportional to household’s total expenditure.

In this study, we applied income quintiles (income groups) and educational level (educational groups) as separate indicator variables for SES. The main reason for choosing these evident indicators was availability of related data in HIES. To evaluate income quintiles, two variables of weighted gross cost decile were used as the proxy variables for provincial and national income quintiles. One variable assessed each household’s income decile within sample households in the same province. The other variable assessed each household’s income decile among all sample households at national level. Based on income deciles, the households were divided into five income quintiles (groups), including the first and second income deciles: households with the lowest income (first income group),the third and fourth income deciles: low-income households (second income group), the fifth and sixth income deciles: middle-income households (third income group), the seventh and eighth income deciles: high-income households (forth income group), and the ninth and tenth income deciles: households with the highest income (fifth income group). Also, to evaluate educational levels, the households were divided into five groups according to the educational level of the head of the household as follows: illiterate or uneducated (first educational group), primary education (second educational group), secondary education or incomplete high school education (third educational group), diploma (forth educational group), and academic education (fifth educational group).

In the final stage, inequalities in household’s dental care expenditure in absolute and relative measures according to income quintiles and educational level were quantified based on the Gini coefficient in addition to the concentration index (CI) [19, 20].

The Gini coefficient has been used mostly for quantifying inter-individual health inequalities [21]. This coefficient is based on the Lorenz curve, where the horizontal axis represents the cumulative proportion of individuals by value of health indicator (here, dental care expenditure), ranked in increasing order. A Gini coefficient of zero indicates perfect equality, where all values are the same, and the Gini coefficient next to 1 indicate great inequality among values. CI is a relative measure of inequality, and it emerged as one of the most common measures to summarize health inequality in a series of subgroups with a natural ordering [20]. The CI has a negative value when the health indicator is concentrated among the disadvantaged (here, the households with the lowest income, and households with a head who was illiterate or uneducated); and it has a positive value when the health indicator is concentrated among the advantaged, (here households with the highest income, and households with a head who had academic education. When there is no inequality, the CI is 0 [22].

## Results

The results showed that about 8% of households had paid for dental care during the month before sampling. Tables 1 and 2 present the descriptive statistics of dental care expenditure in total sample households and also in income and educational groups. The first group corresponded to households with the lowest-income/lowest education in the sample and the fifth group belonged to the households with the highest income/highest education. On average, the household expenditure per capita on dental care was estimated to be higher in groups with higher incomes as well as those households with more educated head. Most of the dental cares paid for including visits, extraction, scaling, dental surgery, and root canal treatment were reported to be 93% and the least dental cares paid for were related to the orthodontic treatments.

**Table 1:** Descriptive statistics of dental care expenditure per capita

Variable	Number of observations	Mean expenditure per capita (in Dollars)	SD
Visits, extraction, scaling, dental surgery, root canal treatment	571	144.21	290.68
Periodontal surgery, dental implants and prostheses	35	554.35	229.07
Orthodontic services	8	628.09	115.71

**Table 2:** Descriptive statistics of dental care expenditure per capita based on income, and educational groups

Income/educational groups	Dental care expenditure subcategories	Mean expenditure per capita (in Dollars)	
		Income groups	Educational groups
First group	Visits, extraction, scaling, dental surgery, root canal treatment	4.35	5.48
	Periodontal surgery, dental implants and prostheses	0	0.36
	Orthodontic services	0	0
Second group	Visits, extraction, scaling, dental surgery, root canal treatment	5.10	9.81
	Periodontal surgery, dental implants and prostheses	0.08	0.51
	Orthodontic services	0.07	0
Third group	Visits, extraction, scaling, dental surgery, root canal treatment	8.34	16.53
	Periodontal surgery, dental implants and prostheses	2.13	1.72
	Orthodontic services	0	0.53
Forth group	Visits, extraction, scaling, dental surgery, root canal treatment	30.40	27.10
	Periodontal surgery, dental implants and prostheses	3.23	8.19
	Orthodontic services	0.93	2.12
Fifth group	Visits, extraction, scaling, dental surgery, root canal treatment	46.93	39.05
	Periodontal surgery, dental implants and prostheses	19.47	17.45
	Orthodontic services	5.63	4.97

Table 3 shows the results of the Gini coefficient (at 95% confidence level) for the dental care expenditure per capita subcategories and total health care expenditure per capita category.

**Table 3:** The Gini index for expenditure

Dental care	Index value	
	Absolute Dental expenditure per capita	Relative Dental expenditure per capita
Total dental cares	0.97	0.96
Visits, extraction, scaling, dental surgery, root canal treatment	0.61	0.98
Periodontal surgery, dental implants and prostheses	0.57	0.99
Orthodontic services	0.59	0.99
Total health care	0.63	0.76

**Fig. 1:**
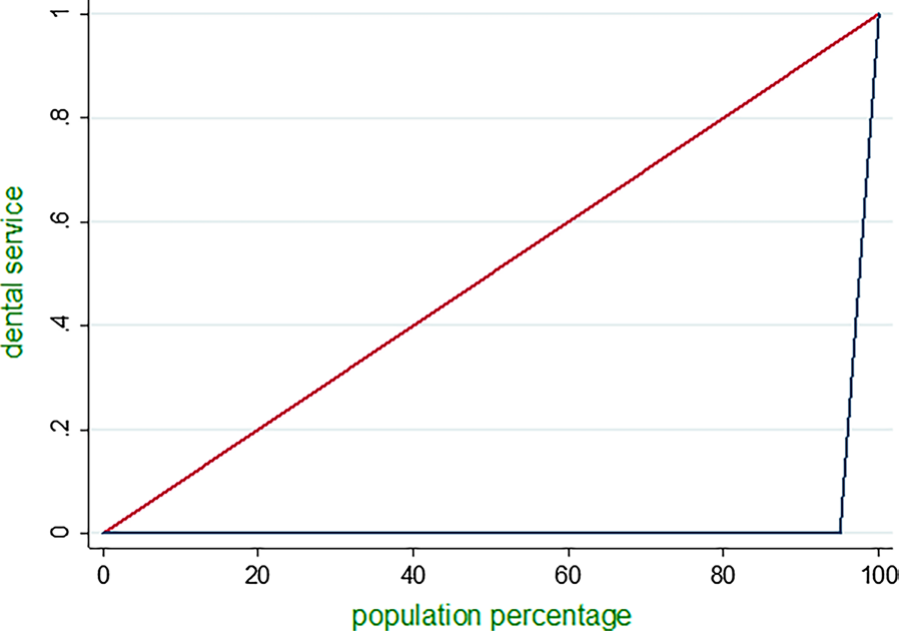
Gini coefficient of dental care expenditure

The Gini coefficient value was estimated to be 0.97 and 0.96 (P value = 0.05) for dental care expenditure per capita respectively in absolute, and relative measure. The indices indicated a significant inequality in these expenditure among the sample households. The extracted Lorenz curves also demonstrated these inequalities (Fig. 1).

Comparison of the Gini coefficient values indicates that inequalities in absolute and relative dental care expenditures were higher than those in expenditure for total health care.

**Fig. 2:**
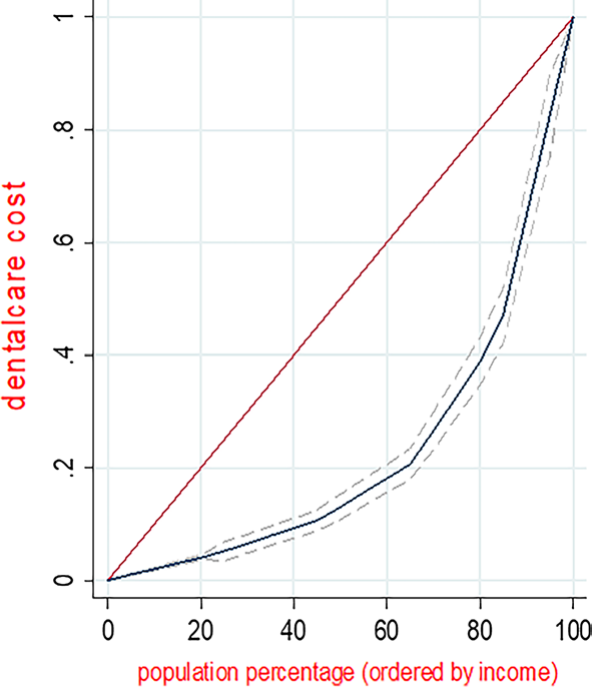
Concentration curve of dental care expenditure (based on income quintiles)

Table 4 shows the CI values for dental care expenditure in respect to provincial and national income quintiles. The values of CI were positive and significant for all dental care subcategories in respect to the both provincial and national income quintiles. This finding indicated an inequality in all subcategories of absolute and relative dental cares expenditure in favor of higher income quintiles. Also, the inequality levels in respect to provincial income quintiles were higher than those in respect to the national income quintiles (Fig. 2).

**Table 4:** CIs for dental care expenditure by the service type in respect to the income quintiles

Dental care	CI in respect to national income quintiles (p-value)		CI in respect to provincial income quintiles (p-value)	
	Absolute Dental expenditure per capita	Relative Dental expenditure	Absolute Dental expenditure per capita	Relative Dental expenditure
Visits, extraction, scaling, dental surgery, root canal treatment	0.47(< 0.001)	0.36 (< 0.001)	0.63 (< 0.001)	0.36 (< 0.001)
Periodontal surgery, dental implants and prostheses	0.70 (< 0.001)	0.37 (< 0.001)	0.82 (< 0.001)	0.61 (< 0.001)
Orthodontic services	0.76 (< 0.01)	0.39 (< 0.001)	0.88 (< 0.01)	0.68 (< 0.001)
Total dental cares	0.54 (< 0.001)	0.32 (< 0.001)	0.69 (< 0.001)	0.38 (< 0.001)

**Fig. 3:**
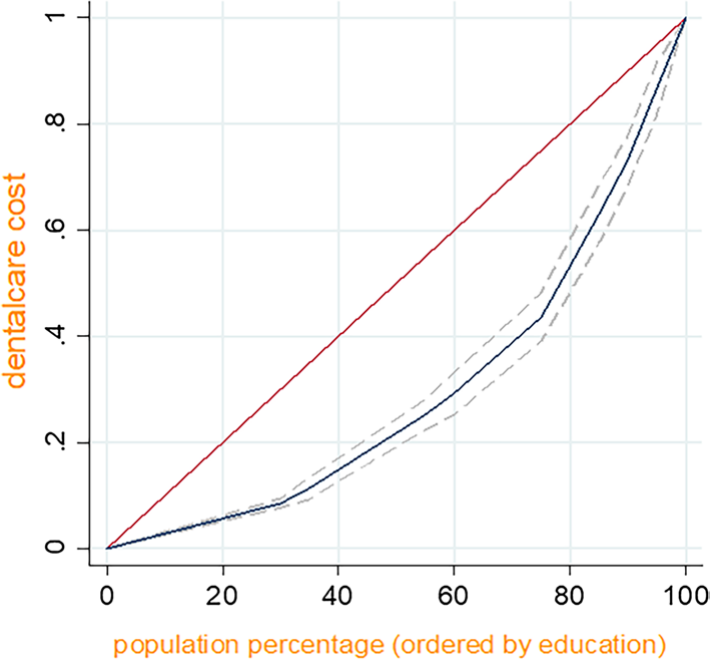
Concentration curve of dental care expenditure (based on education quintiles)

Table 5 shows the CI values for the expenditure of dental care subcategories in respect to the educational level of the head of the household. The values of CI were positive for all dental care subcategories which showed an inequality in absolute and relative dental care expenditure in respect to educational level of the head of the household. Inequality in all the subcategories of dental cares was statistically significant in favor of those with higher educational levels (Fig. 3).

**Table 5:** CIs for dental care expenditure in respect to the educational level of the head of the household

Dental care	CI (p-value)	
	Absolute Dental expenditure per capita	Relative Dental expenditure per capita
Visits, extraction, scaling, dental surgery, root canal treatment	0.37 (< 0.001)	0.27 (< 0.001)
Periodontal surgery, dental implants and prostheses	0.67 (< 0.001)	0.59 (< 0.001)
Orthodontic services	0.75 (< 0.01)	0.73 (< 0.001)
Total dental cares	0.40 (< 0.001)	0.28 (< 0.001)

## Discussion

This study was aimed to investigate inequality in dental care expenditure in Iranian households. Results of the study indicated inequality in absolute and relative measures of total dental cares expenditure and its subcategories between the income and educational groups.

Early findings of the study showed a considerable part of dental care expenditure for households was for the restorative dental care category of extraction, scaling, dental surgery, and root canal treatment [23]. Results of similar studies have also demonstrated that the restorative dental care is the most common reason for referral and expenditure. The studies by Rezaei et al. as well as Abbasi and Haghgoo reported 45% and 71% of the patients had used restorative dental care, respectively [24]. In developed countries, these types of cares also account for a considerable part of dental care expenditure and many citizens of these countries are willing to pay for these cares [23]. The least common reason for dental care expenditure was orthodontics. In the study by Daryazadeh et al., the least common reason for the patients referring to a dental clinic in Isfahan was for orthodontic services, which was consistent with the results of this study. It give the impression that high cost of orthodontic service, lower needs, as well as lack of insurance coverage for these dental services are the reasons for the low use of such services and therefore lower related per capita expenditure [25].

Calculation results of the Gini coefficients showed that inequality in expenditure for dental care in Iranian households was higher than the total health care. The finding demonstrated dental care expenditure were distributed in a more unbalanced manner than total health services expenditure among the households. This finding could indicate the role of dental care expenditure in causing financial difficulty in Iranian households. Some of the previous studies have also suggested this matter. Bernabe et al. (2017) conducted a study on 184,257 people aged 18 years old and more in 40 low- and middle-income countries and reported dental care increased the risk of catastrophic health costs among the poor. They also represented the dental care costs were 1.88 times more catastrophic than total health services costs and were 1.65 times more associated with the probability of poverty than the total health care costs [26]. Richard (2010) estimated the payment for the retired and showed the average payment for dental care for a two-person family would be about 2% of the annual income [27].

Results of the CIs reported an income inequality in dental care expenditure and the subcategories of this expenditure in favor of higher income quantiles. This finding was consistent with those of other studies. The scientific evidence indicated that, when access to health care services depended on the payment ability, the use of dental health service would be limited. Kailembo et al. (2018) showed that although poor people usually experience a different level of oral diseases, this group of people are less likely to refer to a dentist due to the financial obstacles [28]. In contrast, with increasing household income, the probability of referring to a dentist increases and dental expenditure also increase by 31.8% [29]. Also, findings of Nahvi et al. (2017) on a sample consisting of 305 individuals referring to dental clinics in city of Ramsar showed that high-income people had a higher ability to pay for health services [24]. Results of the study by Rezaei et al. (2016) on a sample consisting of 520 household heads indicated a positive relationship between household’s income and use of dental care [30]. In addition, the study by Homaierad et al. (2016) in Shiraz reported people who had higher income used more dental care and the poor did not have the financial ability to use such services [18]. Grytten and Holst (2002) found positive correlation between demand for dental care and income in Norway [31]. In a study conducted in Santa Maria, Brazil, Piovesan et al. (2011) found that children with low SES used fewer dental care [32]. However, a study by Stella et al. (2001) showed a significantly positive relationship between low income and demand for preventive dental care [33] and the results of that study were not consisted with our research. Still, most studies have confirmed that dental expenditure and demand for dental care are less in low-income groups.

Inequality in dental expenditure becomes more important when empirical evidence is taken into account, which shows that the poor have more needs for dental care. A statistically significant relationship between wealth status and the decay-missing-filled (DMF) index has been confirmed in the studies conducted in developing and developed countries. For example, Kazemi et al. (2019) showed that the prevalence of high DMF score among the poorest children was 2.33 times higher than that among the richest children [34]. Results of the study by Kazeruni et al. (2005) also showed that low socio-economic status (SES) was an important factor in tooth decay and was related to high DMF score in Iran [35]. Findings of Pothidee (2016) in Thailand, Martins (2015) in Brazil, and Moradi (2017) in Kurdistan Province, Iran, found a positive relationship between lower SES and poor oral health [36–38]. As the existing evidence shown, lower income classes may have a greater need for dental care services. And as we shown, lower income groups pay less for such services. The reason could be an inability to pay for dental service expenditure. Therefore, the dental health status of poor people could continue to worsen due to lack of financial access to dental care services.

Findings of this study also revealed an inequality in total dental cares expenditure and subcategories of these expenditure in respect to the educational levels. This inequality was in favor of households, in which the head had a higher level of education. Vaal (2012) examined the relationship between education and use of dental care and concluded that higher education had a positive relationship with the use of dental care. In fact, people with higher education had higher awareness of the benefits of dental care and, thus, they use more services [39]. Another study showed that the households with more highly educated members generally spent more for dental scaling. The higher-educated households had higher dental information than other households and had better understating of importance and efficiency of spending money on dental care than others. Hence, the use of these health cares was a higher priority in their preferences. Therefore, these households spent more for dental care [29].

Educational inequality in dental expenditure is important, because results of previous studies have shown that people with higher level of education have lower need for dental cares due to having a higher health level. Results of the study by Okullo et al. (2004) in Ogando showed that students with higher parental educational level were dealing with less dental problems [40]. The study by Kazemi (2019) on 1457 students aged 12–15 years old in Kurdistan Province, Iran, showed that weak DMF index was associated with lower levels of parental education and the weak DMF index among school children, the parents of whom had an academic degree was about 2.27 times lower than the children, the parents of whom had no academic degrees [34]. Hernandez-Palacios et al. (2014) found in their study in Mexico that there was a positive relationship between oral health and educational level [41]. Results of Kuhbar et al. (2018) showed that households, the heads of whom had an educational level lower than high-school degree, were less likely to use the orthodontic services and gum surgery than the households, the heads of whom had high-school degree or above [29].

One of the important results of the present study was that income inequality in both total dental care expenditure and all of its subcategories was higher than the educational inequality in the expenditure of these cares. This finding shows that lack of financial ability could lead to the inequality in dental care expenditure, even more than low educational level and awareness of households. Therefore, increased income level of household heads could be more associated with household expenses for dental care than the educational level.

However, oral health inequality is multifactorial, with several contributors such as the affordability and accessibility of healthy dental food, lifestyle, and the opportunity to obtain and finance preventative and therapeutic dental care all having an impact on this inequality. As a result, future research might add to the findings of this study by looking at additional factors that lead to oral health inequalities. This can assist dental health policymakers in developing more comprehensive measures to address oral health inequalities.

## Conclusion

Income and educational inequalities in the absolute and relative amounts of dental expenses for the Iranian households were in favor of higher income groups or higher educational level of household heads. These inequalities were higher in both total dental expenditure and all its subcategories than the educational inequalities of the expenditure. In order to reduce these inequalities, the policymakers need to pay special attention to low-income households, particularly those with low-educated heads.

## Data Availability

Data are available from the authors upon reasonable request and with permission of the Statistical Center of Iran.
